# Epidemiological Study of Stroke Presenting to the Emergency Department of a Tertiary Hospital: An Observational Study

**DOI:** 10.31729/jnma.9023

**Published:** 2025-05-31

**Authors:** Bibek Rajbhandari, Yogendra Man Shakya, Ramesh Kumar Maharjan, Yagya Laxmi Shakya, Shiva Sharma Aryal, Pratiksha Bhandari, Ram Prasad Neupane, Olita Shilpakar, Rajan Narayan Nakarmi, Cimona Shrestha

**Affiliations:** 1 Department of General Practice, Maharajgung Medical Campus, Institute of Medicine, Maharjgunj, Kathmandu, Nepal; 2 Department of Emergency Medicine, Maharajgung Medical Campus, Institute of Medicine, Kathmandu, Maharjgunj, Kathmandu, Nepal; 3 Department of Nursing, Patan Academy of Health Sciences, Lagankhel, Lalitpur, Nepal

**Keywords:** *emergency department*, *stroke*, *Nepal*

## Abstract

**Introduction:**

Stroke remains a major global health burden, ranking as the second leading cause of death and third leading cause of disability worldwide. Low- and middle-income countries, including Nepal, face a disproportionate share of this burden, characterized by delayed treatment and limited healthcare infrastructure. This study aimed to determine the epidemiological profile of stroke cases presenting to the emergency department of a tertiary hospital in Nepal.

**Methods:**

A cross-sectional study was conducted using medical records of stroke patients presenting to the Emergency Department from August 2022 to September 2023. Data on demographics, stroke type, clinical outcomes, and management were collected and analyzed descriptively using STATA version 17.

**Results:**

Of 39,702 emergency department visits, 1,174 (2.96%) were stroke cases. Ischemic stroke occurred in 896 (76.34%) patients, hemorrhagic stroke in 175 (14.89%), and transient ischemic attack in 103 (8.82%). The mean age was 61.79 ± 15.76 years, and 704 (60.00%) patients were male. Admission to the general ward occurred in 1,011 (86.10%) patients, and 43 (3.65%) patients were admitted to the ICU. Thrombolysis was administered to 22 (1.87%) patients, and 11 (0.93%) were referred for thrombectomy. A total of 151 (12.90%) patients arrived within 4.5 hours of symptom onset.

**Conclusions:**

The study highlights the predominance of ischemic stroke, delayed hospital presentations, and limited use of thrombolysis among stroke patients in a high-volume ED in Nepal.

## INTRODUCTION

Stroke is the second leading cause of death and the third leading cause of disability worldwide, with an estimated 12.2 million new cases, 143 million disability-adjusted life years (DALYs) lost, and 6.6 million deaths in 2019.^1^ Over the past three decades, the number of stroke cases, prevalence, and related deaths have risen significantly. Low- and middle-income countries (LMICs) bear a disproportionate burden, accounting for 70% of strokes and 87% of stroke-related deaths and DALYs.^2^ In LMICs, strokes occur at younger ages and are often hemorrhagic, worsened by delayed treatment and limited healthcare infrastructure. Nepal reported a relatively high crude and age-standardized prevalence of stroke in the southwestern region in 2018, with rates of 2368 and 2967 per 100,000 population, respectively.^3^ The emergency care system in Nepal faces significant challenges, including inadequate training of healthcare personnel and a lack of organized stroke care protocols.^4^

A detailed understanding of stroke epidemiology in emergency settings is crucial to improving outcomes. This study aimed to determine the epidemiological profile of stroke cases presenting to the emergency department.

## METHODS

This study is part of a larger cohort investigation aimed at understanding the relationship between hyperglycemia and morbidity and mortality outcomes in patients with acute stroke. Specifically, this sub-study focuses on the impact of varying glycemic levels on short-term (1 month), midterm (3 months), and long-term (1 year) mortality rates.^5^ Additionally, a cross-sectional study was conducted to analyze the epidemiological profile of stroke cases within the same patient cohort.

The study was conducted at the Emergency Department of Teaching Hospital, a high-volume facility that serves a diverse patient population, including a significant number of individuals presenting with acute stroke symptoms. The study period spanned from August 2022 to September 2023, encompassing a full year of stroke-related cases. All patients diagnosed with stroke who presented to the General Practice and Emergency Department during this time frame were included in the analysis. No sampling technique was employed, as the study aimed to include all eligible cases to ensure comprehensive data collection and representation of the acute stroke patient population.

Data were extracted from the medical records of patients diagnosed with stroke using a structured data collection tool designed to capture key clinical and demographic information. The following variables were included in the dataset: age, sex, type of stroke (ischemic or hemorrhagic), admission to the Intensive Care Unit (ICU), admission to the general ward, admission to the High Dependency Unit (HDU), instances of patients leaving against medical advice, cases of mortality within the emergency department, discharge upon request, thrombolysis administration, and referrals for thrombectomy. These variables were selected to reflect the epidemiological characteristics of stroke patients and the immediate clinical outcomes following their presentation to the emergency department.

The data were recorded electronically using the KOBO Toolbox platform, which provided a user-friendly interface for efficient and accurate data management. Once data collection was completed, the dataset was exported to STATA version 17 for statistical analysis.

Descriptive statistical methods were employed to summarize the data, with frequencies and percentages used to present categorical variables. The 95% confidence intervals (CIs) around proportions were calculated using the Clopper-Pearson exact method to account for variability and provide more accurate estimates of the population parameters. The analysis was designed to identify key trends and associations in the epidemiological profile of acute stroke cases during the study period.

Formal permission to access and utilize the medical record data for research purposes was obtained from the hospital administration. The study was reviewed and approved by the Institutional Review Board (IRB), with ethical approval granted under reference number 6-11E2. All procedures followed were in accordance with the ethical standards established by the IRB, ensuring the confidentiality and privacy of patient information.

## RESULTS

The prevalence of stroke among emergency department visits was 1174 (2.96%). Ischemic stroke occurred in 896 (76.34%), hemorrhagic stroke in 175 (14.89%), and transient ischemic attack in 103 (8.82%). The mean age of stroke patients was 61.79± 15.76 years, and 704 (60%) were male.

**Table 1:** Epidemiological profile of stroke cases in ED (n=1177).

Epidemiological profile	Total n(%)	IS n(%)	HS n(%)	TIA n(%)
Admitted to ICU	43(3.65)	10(23.26)	33(76.74)	0(0.00)
Left against medical advice	7(0.59)	1(14.29)	5 71.43)	1(14.29)
Admitted to general ward	1014(86.10)	795(78.39)	129(12.72)	90(8.88)
Admitted to HDU	62(5.27)	52(83.87)	3(4.84)	7(11.29)
Mortality in the emergency department	5(0.42)	1(20)	3(60)	1(20)
Discharge upon request	13 (1.1)	6(46.15)	2(15.38)	5(38.46)
Thrombolysis	22(1.87)	22(100)	0(0)	0(0)
Referral for Thrombectomy	11(0.93)	11(100)	0(0)	0(0)
Duration of Time Visiting Hospital After Onset of Symptoms				
4.5 hours	153(12.9)	99(12.4)	50(11.3)	35(15.3)
>4.5hours	1024(87.2)	799(87.6)	125(88.7)	69 (84.7)

* IS: Ischemic Stroke; HS: Hemorrhagic Stroke; TIA: Transient Ischemic Attack

This epidemiological profile of 1177 stroke cases in the emergency department provides insights into stroke management and outcomes. ICU admission was reported in 43 (3.65%), discharge against medical advice in 7 (0.59%), general ward admission in 1014 (86.10%), and HDU admission in 61 (5.18%). Mortality in the emergency department occurred in 5 (0.42%) cases, and discharge upon request was reported in 13 (1.10%) cases. Thrombolysis was administered in 22 (1.87%) cases, and 11 (0.93%) were referred for thrombectomy. A total of 152 (12.91%) patients arrived within 4.5 hours of symptom onset, while 1025 (87.13%) arrived after 4.5 hours (Table 1).

## DISCUSSION

This study highlights the epidemiological characteristics and early clinical outcomes of stroke cases presenting to a high-volume emergency department in Nepal. Among 39,702 emergency visits over one year, the prevalence of stroke was 2.96%. Ischemic stroke was found to be the most prevalent type, accounting for 76.34% of cases. This is consistent with reports from Western countries and Arab countries, where ischemic stroke is significantly more common than hemorrhagic stroke.^6-8^ A systematic review assessing the status of stroke care in Nepal also found that ischemic stroke was more frequent (70.87%) than hemorrhagic (26.79%).^9^ These parallels suggest that despite regional and resource differences, the underlying epidemiology of stroke subtypes remains broadly similar, underscoring the need for robust systems capable of managing ischemic stroke, including timely access to imaging, thrombolysis, and secondary prevention services.

The epidemiological profile of stroke cases in our study revealed that 3.06% of patients required admission to the Intensive Care Unit (ICU). In contrast, global estimates suggest that between 10% and 20% of patients with acute stroke necessitate ICU care^10-12^. This significant discrepancy may be attributed to Nepal's status as a low-resource country, where the availability of ICU beds is limited. Additionally, many patients face substantial out-of-pocket expenses for healthcare services, making ICU charges unaffordable for a large population segment. Consequently, due to both the scarcity of ICU resources and the financial burden associated with their use, many stroke patients are instead managed in general wards. Approximately 85% of stroke patients in our study were admitted to these less intensive care settings, highlighting the challenges faced by the healthcare system in providing adequate critical care for stroke patients.

Thrombolysis was administered to only 1.9% of stroke cases in our study, a rate significantly lower than those reported in other countries such as Qatar (12.5%) ^8^, North America (8.29.4%) ^13^, and Sweden (6.7%) ^14^. A systematic review highlighted considerable variability in thrombolysis rates across Asia, with high-income countries (HICs) averaging 11.3% compared to just 8.1% in low- and middle-income countries (LMICs). This is far below the 50% target the American Stroke Association set for thrombolysis administration in eligible patients. In our setting, the main factor contributing to the low rate of thrombolysis was the delays in seeking hospital care, as intravenous thrombolysis (IVT) can only be administered within a 4.5-hour window from the onset of symptoms. Only 12.9% of stroke patients arrived at the hospital within this critical time frame. Studies have identified key barriers to achieving higher thrombolysis rates in LMICs, including a lack of human resources, limited accessibility to healthcare facilities, and the high costs associated with standard thrombolysis treatments.^15,16^ These factors often deny many patients the opportunity for intravenous thrombolysis (IVT), particularly in settings where insurance systems are inadequate or nonexistent.^15^ In response to these systemic challenges, a notable step was taken by the Government of Nepal in 2025, which introduced the provision of free thrombolytic drugs through four major hospitals.^17^ While this is a commendable initiative, broader efforts are needed. Public education campaigns to improve symptom recognition, faster emergency medical services, and expansion of stroke-ready centers are critical to improving thrombolysis rates and overall stroke outcomes in Nepal.

Additionally, referral for thrombectomy was observed in only 0.93% of cases in our study population. Historically, thrombectomy was not available at our institution, which is one of the largest tertiary care hospitals in Nepal. This reflects a broader national limitation in access to endovascular stroke interventions. However, the recent introduction of thrombectomy services at our center in 2025 represents a significant milestone in enhancing stroke care capacity. As this high-impact neurointervention becomes more accessible, it is imperative to establish clear referral pathways, develop endovascular expertise through targeted training programs, and integrate thrombectomy into standardized stroke care protocols. These measures are essential for the timely identification and management of eligible large vessel occlusion (LVO) strokes, ultimately contributing to improved clinical outcomes.

One of the major strengths of this study is its large sample size, encompassing emergency department visits over a one-year period, which enhances the generalizability of the findings within the hospital catchment area. By including all diagnosed stroke cases during this timeframe, the study provides a comprehensive overview of the burden and clinical profile of stroke in a high-volume, real-world emergency care setting in Nepal. The study also reflects routine clinical practice, capturing data on presentation times, management strategies, and immediate outcomes, which are important for informing local health policy and emergency care improvements.

While this study has several strengths, it also has notable limitations. This study has several limitations that should be considered when interpreting the findings. First, it was a single-center study conducted in a tertiary care hospital, which may limit the generalizability of the results to other healthcare settings, particularly rural or primary care centers in Nepal. Second, the study was based on retrospective review of medical records, and data quality depended on the completeness and accuracy of documentation, which may have introduced information bias. Third, the study did not assess functional outcomes (such as disability status) or long-term follow-up of stroke patients, restricting the understanding of stroke recovery and prognosis.

## CONCLUSION

This study provides a comprehensive overview of the epidemiological profile of stroke cases presenting to the emergency department of a tertiary hospital in Nepal. The findings highlight the predominance of ischemic stroke and the significant delays in patient presentation, with most patients arriving after the critical 4.5-hour window for thrombolysis administration.
